# Synthesis of giant panda habitat suitability evaluations

**DOI:** 10.1016/j.heliyon.2024.e37398

**Published:** 2024-09-04

**Authors:** Guanyu Mu, Xiaotong Shang, Han Pan, Tao Ruan, Biao Yang, Li Zhang

**Affiliations:** a Key Laboratory for Biodiversity Science and Ecological Engineering, Ministry of Education, College of Life Sciences, Beijing Normal University, Beijing, 100875, China; b College of Life Science, China West Normal University, Nanchong, 637002, China; c Society of Entrepreneurs and Ecology (SEE) Foundation, Beijing, 100012, China; d Chengdu Aisiyi Ecology Conservation Centre, Chengdu, 610000, China

**Keywords:** Habitat ecology, Models, Indicators, Wildlife protection

## Abstract

Degradation, fragmentation, and habitat loss significantly threaten the survival of giant pandas (*Ailuropoda melanoleuca*). Habitat suitability evaluations (HSEs) represent a crucial component of giant panda habitat research. However, a systematic review of HSE research on giant pandas has not been conducted in recent years. To make up for that, we synthesised HSE research on giant pandas publicated from 2013 to 2022 and conducted a comprehensive analysis of the evaluation scale, evaluation methods, and research findings. We found a correlation between the geographical distribution of giant pandas and HSE-based studies on giant pandas. Furthermore, we observed a trend towards interdisciplinary and large-scale research. Although the evaluation accuracy has improved compared to that of earlier work, some limitations still remain, such as concentrated evaluation areas, incomplete indicators, and outdated data. Current HSE research on giant pandas helps determine suitable habitat distributions, facilitating protection strategies and management planning for protected areas. We suggest that future research should prioritize those unexplored or under-evaluated areas, incorporate a broader range of microenvironmental indicators, and update data resources and methodologies. This study bridges the gap in systematic reviews on HSEs of the giant panda and provides valuable references and recommendations for future research as well as the protection and management of giant panda habitats.

## 1 Introduction

A biological habitat is composed of biotic and abiotic environments and refers to sites where organisms live and reproduce [1]. According to the Living Planet Report 2018, the World Wildlife Fund (WWF) reported a 60 % decline in wild animal populations on Earth since 1970. The main reasons for this decrease are habitat loss, fragmentation, and degradation [2]. Habitat ecology is the key field in ecological research. Habitat quality refers to the ability of an ecosystem to promote the sustainable development of individuals and populations within a certain spatio-temporal range, and it is the basis of ecosystem service functions and an important factor affecting biodiversity [3]. Habitat suitability evaluation (HSE) is an effective method of assessing the quality of existing habitats of wild animals. The process involves determining evaluation indices based on habitat selection theories of the target species, collecting relevant data, and selecting suitable models for analysis. A habitat suitability distribution map of the target species is then obtained, and the habitat quality distribution conditions in the study area are revealed [4]. These results can guide the restoration of degraded habitats and inform the planning and management of protected areas.

The giant panda (*Ailuropoda melanoleuca*) is a rare and endangered species unique to China [5]. Historically, giant pandas were widely distributed in the Yellow, Yangtze, and Pearl River Basins. However, climate change and human activities have led to their extinction in many regions [6]. Now they can only be found in the forested mountain ranges of the Qinling, Minshan, Qionglai, Xiaoxiangling, Daxiangling, and Liangshan mountains [7]. To protect and restore the ecological environment, China has implemented several ecological projects, such as the Natural Forest Protection Program, Grain to Green Program, and Wildlife Conservation and Nature Reserve Construction Program. These projects have played a positive role in protecting and restoring the habitats of giant pandas [8]. In 2016, the International Union for Conservative of Nature (IUCN) downgraded the extinction risk level of giant pandas from ‘endangered’ to ‘vulnerable’, reflecting the initial success of giant panda protection. However, almost all HSE studies have shown that giant panda habitats are severely fragmented [9]. Degradation and fragmentation caused by human or natural interference remain the main threats to the survival of wild giant pandas [10].

Habitat ecology is a crucial field in giant panda research, focusing on the composition, structure, and function of their habitats [11]. HSEs are a key aspect of this research, exploring habitat quality and spatial patterns [8,9]. To establish more effective protective measures for giant pandas, the distribution and quality of their habitats must be better comprehended [12]. Research HSEs for giant pandas began in the 1990s [13]. Early research primarily used human experience to assign weights to factors and construct simple mechanistic models. Scores for different factors were summed for each geographic cell to create an overall habitat suitability distribution map [1,14,15]. These early studies were characterised by small scales, incomplete evaluation indicators, outdated data, strong subjectivity, lack of empirical evidence, and large errors [16]. In addition, they focused more on qualitative or quantitative descriptions of habitat attributes and lacked in-depth assessments of the correlation between landscape patterns and ecological processes [9].

In recent years, ecological niche models, which can reflect the quantitative relationship between habitat suitability and environmental factors more objectively than traditional mechanism models, have been widely applied to research on giant panda HSEs [[17], [18], [19]]. With the advancement of “3S″ technology (Geographic Information System, Remote Sensing, Global Position System), now habitat quality can be studied at the landscape level. Additionally, there is a trend towards larger spatial and temporal scales in HSE research [12,20]. Habitat suitability assessment techniques for giant pandas have diversified, expanding from an early focus solely on habitat quality [1,14,21] to the inclusion of factors such as climate change [22,23], same-domain species relationship [24,25], reserve design and management [26], and habitat corridor construction [27,28].

Over the past 10 years, several reviews have been published on researches of giant pandas and their habitats, covering habitat selection [29], giant panda ecology and protection [8], natural interference [30,31], human interference [32], and habitat restoration [11]. However, a systematic review of HSE-based research on giant pandas has not yet been conducted. This study reviewed the literature on giant panda HSEs from 2013 to 2022 and summarised the main research progress to address the following questions: (1) At which scale (temporal, spatial, research fields involved) was the evaluation conducted? (2) How did researchers conduct evaluations (models, data source, indicators)? (3) What guidance and suggestions the evaluation could provide for protecting giant pandas?

## 2 Methods

A search was conducted for papers published in English-language journals indexed in the Web of Science (WOS) database, with ‘giant panda’ in the ‘title’ item and ‘habitat evaluation’ or ‘habitat assessment’ in the ‘topic’ item (title, abstract, author keywords, and keywords plus). A search was performed for papers published in journals in English (Web of Science, WOS) using the words ‘giant panda’ in the ‘title’ item and ‘habitat evaluation’ or ‘habitat assessment’ in the ‘topic’ item (title, abstract, author keywords, and keywords plus). The publication date was set within the period from January 1st, 2013 to December 31st, 2022. The search was conducted in ‘all databases’.

Given that many papers on giant pandas are written in Chinese, we conducted an additional search for papers published in Chinese journals using the China National Knowledge Infrastructure (CNKI), using the words ‘giant panda’ in the ‘title’ item and ‘habitat evaluation’ or ‘habitat assessment’ in the ‘title, keyword and abstract’ item. The publication date was set within the period from January 1st, 2013 to December 31st, 2022, and ‘all journals’ was selected as the source type. To avoid the duplication, only Chinese-language papers were retained from the CNKI search results.

We included articles that used HSEs techniques in research. Ultimately, 43 qualified articles were selected (Table S1).

After retrieval, a statistical analysis was performed on the research sites, fields involved, time scales, model use, data sources, and indicator selection from these documents.

## 3 Results

### 3.1 Research scale

#### 3.1.1 Spatial scale

**Fig. 1:**
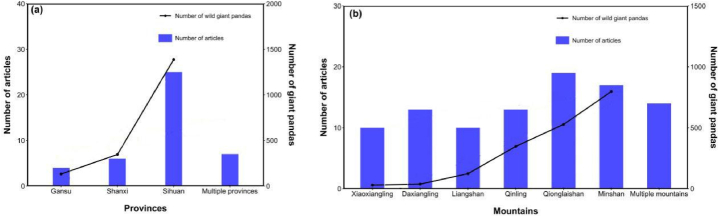
Geographical distribution of study areas of giant panda HSE research: (a) Provinces and (b) Mountains.

An analysis of giant panda HSE research areas revealed that at the provincial scale, most studies focused on Sichuan Province (62.8 %, 27 articles), while fewer studies were conducted in Shanxi Province (14.0 %, 6 articles) and Gansu Province (9.3 %, 4 articles) (Fig. 1a). Regarding mountain ranges, the highest number of studies focused on Qionglaishan (48.8 %, 21 articles) and Minshan (39.5 %, 17 articles), and several studies conducted multiple mountain evaluations (34.9 %, 15 articles) (Fig. 1b).

More than half of the studies evaluated only one or few reserves (within 5) (22 articles, 51.2 %), within which were evaluated multiple times being Wolong (9.3 %,4 articles), Wanglang (7.0 %,3 articles), Fengtongzhai (7.0 %,3 articles), Baishuijiang (7.0 %,3 articles), Tangjiahe (4.7 %,2 articles), Foping (4.7 %,2 articles), Dafengding (4.7 %,2 articles), Labahe (4.7 %,2 articles), and Daxiangling Nature reserve (4.7 %,2 articles).

#### 3.1.2 Temporal scale

Most studies (72.1 %, 31 articles) assessed only the current habitat quality. There were six studies evaluating previous habitat quality, mainly around the year of 2000 (11.6 %, 5 studies). Six studies predicted habitat quality for 2050, and three studies for 2070. Two studies predicted the habitat quality for approximately 2100 (Table S1). Researches predicting the future habitat quality of giant pandas were often combined with climate change investigations. For example, Liu et al. (2016) [23] predicted the suitable habitat distribution for giant pandas in the northern Minshan Mountains for 2050 based on their temperature preferences.

#### 3.1.3 Research fields

We classified and organised 32 papers that combined HSE research with other research fields into nine categories: overlapping analysis of sympatric animals, impacts of human interference, climate change, earthquakes, reserve design and management, overlapping patterns of giant panda resting and path habitats, overall range change of habitat, habitat corridor research, and impact of selecting spatial scales when assessing species habitats and distribution (Table 1).

**Table 1:** HSE researches combined with other research fields.

Fields involved	Number of articles	References
Overlapping analysis of sympatric animals	5	[24,25,64,73,74]
Human interference	7	[12,19,24,38,61,71,75]
Climate change	7	[19,20,22,23,48,71,76]
Reserve design and management	7	[26,37,41,51,[77], [78], [79]]
Earthquake	1	[39]
Overlapping patterns of giant panda resting and path habitats	1	[44]
Changes in the overall range of habitats	1	[49]
Habitat corridor research	6	[27,28,42,52,53,72]
Impact of selecting spatial scales when assessing species habitat and distribution	1	[58]
Relationship between habitat quality and ecosystem service value	1	[77]

### 3.2 Evaluation method

#### 3.2.1 Data source

**Fig. 2:**
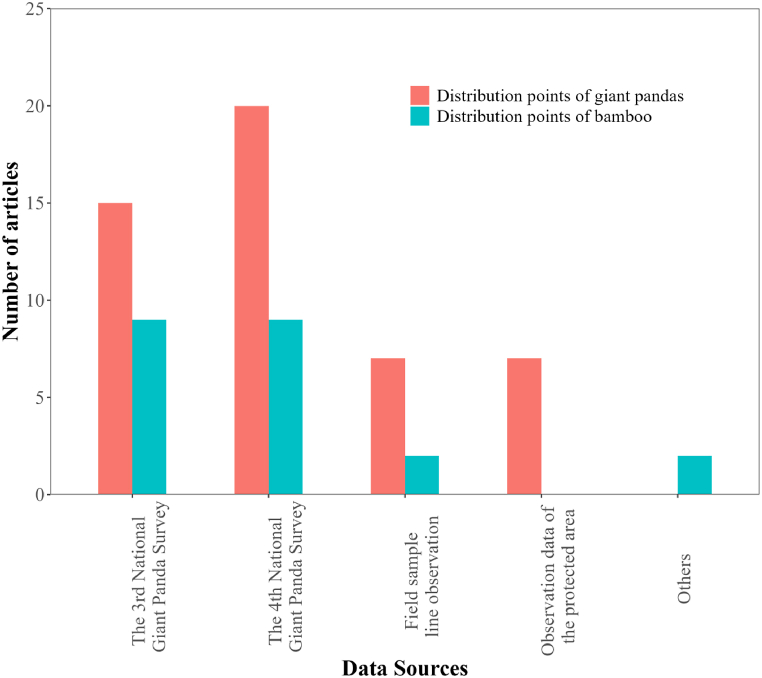
Data sources for giant panda and bamboo distribution points.

We statistically analysed the data sources for giant panda and bamboo distribution points. Most studies obtained the distribution points of giant pandas from the Third and Fourth National Giant Panda Surveys (34.9 %, 15 articles; 46.5 %, 20 articles; respectively). Fewer studies obtained the distribution points of giant pandas from observational data from reserves (16.3 %, 7 articles) or field sample line observations (16.3 %, 7 articles) (Fig. 2). Bamboo distribution data were also primarily from the Third and Fourth National Giant Panda Surveys (20.9 %, 9 articles; 20.9 %, 9 articles; respectively) (Fig. 2).

#### 3.2.2 Model use

The evaluation models used in HSE research can categorized into three types:ecological niche models (62.8 %, 27 articles), mechanism models (34.9 %, 15 articles), and regression models (9.3 %, 4 articles). Three articles (7.0 %) employed multiple model types. Ecological niche models utilize mathematical algorithms to correlate species distribution with environmental variables, calculating the probability of species occurrence in the study area [33]. Mechanism models are constructed based on expert knowledge, selecting key ecological factors affecting species survival and assigning weights to each factor using methods such as the analytic hierarchy process [14,34]. Regression models assess the relationship between species distribution information and environmental variables by dividing the study area into several cells and using presence/absence data of the target species from sampled locations as the dependent variable [35,36]. Among ecological niche models, the Maxent model (58.1 %, 25 articles) was the most frequently used, while the ecological niche factor analysis model (ENFA model) was less frequently used (4.7 %, 2 articles). For mechanism models, weights of factors were primarily set using either the analytic hierarchy process (AHP) (16.3 %, 7 articles) or artificial subjective definition (16.3 %, 7 articles).

#### 3.2.3 Indicator selection

**Fig. 3:**
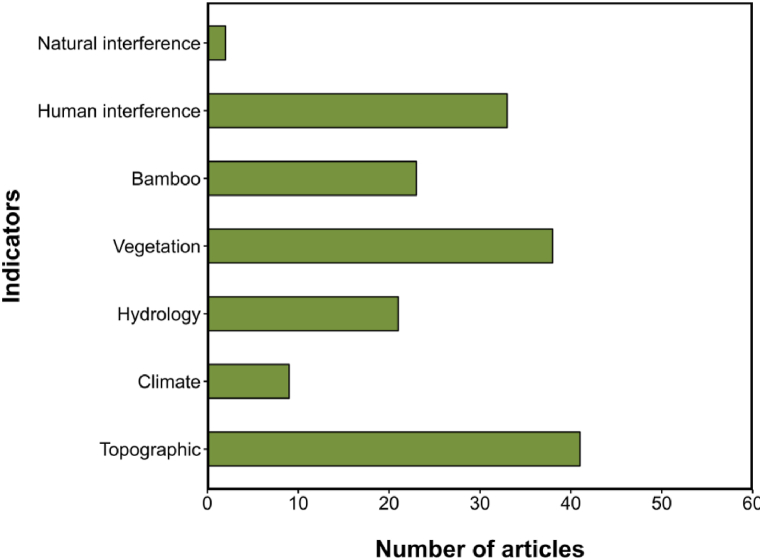
Distribution of selected indicators in HSE research.

We summarised the evaluation indicators selected in the 43 articles and divided them into seven categories (Fig. 3): topography, climate, hydrology, vegetation, bamboo, human interference, and natural interference. Two articles (4.7 %) did not specifically describe the selected indicators. Topography had the highest usage rate at 95.3 % (41 articles). Besides, vegetation (88.4 %, 38 articles), human interference (76.7 %, 33 articles), food resource (bamboo) (53.5 %, 23 articles), and hydrology (48.8 %, 21 articles) were also widely used. Conversely, climate (20.9 %, 9 articles) and natural interference (4.7 %, 2 articles) were less frequently employed. The frequency of indicator usage is detailed in Table 2.

**Table 2:** Statistical analysis of the frequency of indicators usage in 43 articles.

Indicator categories	Indicators	Number of articles (Proportion/%)
Topographic	Altitude	41 (95.3 %)
	Slope	40 (93.0 %)
	Aspect	30 (69.8 %)
	Terrain roughness index, Slope position	2 (4.7 %)
	Shape, solar radiation index	1 (2.3 %)
Climate	Bio12^a^	4 (9.3 %)
	Bio1^b^	3 (7.0 %)
	Bio4, Bio5, Bio10, Bio15, Bio19^c^	2 (4.7 %)
	Bio2, Bio3, Bio6, Bio7, Bio11, Bio14, Bio17, Bio18^d^ Average temperature from May to October, Average temperature in January	1 (2.3 %)
Hydrology	Distance from rivers	14 (34.9 %)
	Distance from water sources	5 (11.6 %)
	Distance from the water system	1 (2.3 %)
Vegetation	Vegetation coverage type	32 (74.4 %)
	Forest coverage rate	6 (14.0 %)
	Net primary productivity	2 (4.7 %)
	Maximum forest patch index, Forest edge density, Forest aggregation index, Enhanced vegetation index	1 (2.3 %)
Food resource	Bamboo species	19 (44.2 %)
	Bamboo coverage	3 (7.0 %)
	Bamboo altitude distribution, Bamboo suitability	1 (2.3 %)
Human interference	Distance from roads	31 (72.1 %)
	Distance from residential areas	17 (39.5 %)
	Distance from agricultural traces	8 (18.6 %)
	Distance from tourist attractions/hydropower stations/	5 (11.6 %)
	Distance from grazing sites/mining traces	4 (9.3 %)
	Distance from medicinal herb picking/bamboo shoot picking traces	3 (7.0 %)
	Distance from logging traces	2 (4.7 %)
	Distance from power transmission lines/medicinal herb picking/fire/hunting/paint collection traces	1 (2.3 %)
Natural interference	Geological hazard density, natural hazard point density	1 (2.3 %)

a Bio12: Annual Precipitation.

b Bio1: Annual Mean Temperature.

c Bio4: Temperature Seasonality, Bio5: Max Temperature of Warmest Month, Bio10: Mean Temperature of Warmest Quarter, Bio15: Precipitation Seasonality.

d Bio2: Mean Diurnal Range, Bio3: Isothermality, Bio6: Min Temperature of Coldest Month, Bio7: Temperature Annual Range, Bio11: Mean Temperature of Coldest Quarter, Bio14: Precipitation of Driest Month, Bio17: Precipitation of Driest Quarter, Bio18: Precipitation of Warmest Quarter.

### 3.3 Protection recommendations

The main challenges in protection of giant pandas habitat include human interference, natural interference, unreasonable planning and design of reserves, and inadequate management by relevant departments. Human disturbances such as grazing and infrastructure construction can affect the habitat utilization patterns of giant pandas, leading to habitat loss, degradation and fragmentation. Consequently, these would cut off population diffusion corridors, isolate small populations, and block gene flow [37,38]. Natural disturbances fall into two main categories: geological hazards and bamboo flowering. Earthquakes and other geological disasters can devitalize wild giant pandas, damage vegetation structures, exacerbate habitat fragmentation, cut off habitat corridors, and block gene flow [39]. Bamboo flowering can lead to widespread death of bamboo which will deprive food resource of giant pandas and exacerbate habitat fragmentation [23,40]. The impact of unreasonable planning and design of reserves can be categorized into three main areas. First, a large number of suitable habitats for giant pandas lie outside reserve boundaries and are inadequately protected [17,20]. Secondly, numerous high-quality habitats within reserves are not included in core areas, risking human disturbance and destruction [26,41]. Thirdly, the design of some reserves overlooks ecological processes, leading to Thirdly, the design of some reserves overlooks ecological processes, leading to isolation of small population [42]. Inadequate management by relevant authorities is mainly due to imperfect legal and regulatory systems, making it difficult for reserve staff to manage the internal environment effectively. Human interference, such as grazing, persists [38].

Regarding protection suggestions, some measures such as controlling human interference (19 articles, 44.2 %), protecting habitat corridors (20 articles, 46.5 %), and restoring degraded habitats (14 articles, 32.6 %) were mentioned the most frequently. Other recommendations include expanding the control scope of reserves (6 articles, 14.0 %), adjusting the planning and management of reserves (8 articles, 18.6 %), strengthening management and monitoring (6 articles, 14.0 %), and improving protection regulations and systems (1 article, 2.3 %).

## 4 Discussion

### 4.1 Research scale

#### 4.1.1 Spatial scale

Our statistics show that the geographical distribution trend in study areas is spatially consistent with that of giant pandas in the Fourth National Giant Panda Survey, with the highest number of study areas in the Minshan Mountains and Qionglaishan Mountains of Sichuan (Fig. 1). Research on giant panda HSE has shown a trend towards large-scale and multiple mountain ranges. Evaluations of multiple mountain ranges accounted for a large proportion. These large-scale studies can guide the protection of giant panda habitats at the landscape level, which was rare in the past [16].

However, many studies have concentrated on individual regions and protected areas, such as Wolong Nature Reserve [39,43], Wanglang Nature Reserve [24,40,44], Baoxing County, Tianquan County, Yingjing County, and Lushan County in the northern part of Ya'an City [19,45,46] (Table S1). According to the Fourth Giant Panda Survey in Sichuan Province, 675,500 hm^2^ of giant panda habitats lie outside protected areas, accounting for 33.32 % of the province's giant panda habitats [47]. However, giant panda habitats outside reserves are often overlooked in HSE studies because of difficulties in data collection and other reasons. Therefore, future research should focus on areas in which HSEs are in blank or limited.

#### 4.1.2 Temporal scale

A significant number of studies have utilized HSE techniques to compare past, present, and future changes in giant panda habitat quality and to predict future habitat distribution, thereby providing recommendations for reserve planning and management [22]. The most common approach is assessments in conjunction with climate change. Climate change is widely recognised as one of the most serious threats to biodiversity, especially for endangered species, such as the giant panda, which has a small population, low genetic diversity, and fragmented habitat distribution [48]. Using HSE techniques, researchers can identify potential future distribution areas for giant pandas under various climate change scenarios. For example, Fan et al. (2014) [20] incorporated climate variables into an assessment model to predict future (2070–2100) changes in the distribution and scope of giant panda habitats under various climate change scenarios.

In addition, the temporal comparison demonstrated that China's giant panda protection efforts have yielded positive results. To illustrate, Yang et al. (2017) [49] assessed the overall range of change in giant panda habitats using data from the Third and Fourth National Giant Panda Survey. The results showed that habitat quality within the entire distribution range of giant pandas has been improved, except for some areas in the Qionglaishan Mountains and southern Minshan Mountains. Yang et al. (2021) [50] compared giant pandas habitat suitability in nature reserves between 2001 and 2013, highlighting the significant role of China's natural reserves in enhancing panda habitat suitability.

#### 4.1.3 Research fields

More than half of the studies (74.4 %, 32 articles) not only focused on evaluating the quality of giant panda habitats, demonstrating a shift from singular HSEs to a more diversified approach. For example, climate data are used to predict the development trend of giant panda habitats under future climate change [20,22,23]. Additionally, HSE models are used to assess the effectiveness of protected areas management [50,51] and to establish locations for panda population diffusion corridors based on suitable habitat distribution patterns [27,28,52,53]. This diversity in research indicates the growing importance of HSEs in understanding and managing giant panda habitats.

### 4.2 Research methods

#### 4.2.1 Data source

The use of outdated and limited experimental data in giant panda HSEs remains a significant issue. Most studies relied on data from the Third and Fourth National Giant Panda Surveys (34.9 %, 15 articles; 46.5 %, 20 articles; respectively). Few studies verified their findings through field visits (Fig. 2). Considering that the Third and Fourth National Giant Panda Surveys were conducted several years ago, the accuracy of evaluating the quality of giant panda habitats based on these data needs verification. Future research should update data resources and data collection technologies should be updated to improve data accuracy.

#### 4.2.2 Model usage

In terms of model use, we found that ecological niche models (ENMs) were used the most (62.8 %, 27 articles), followed by mechanism models (34.9 %, 15 articles) and then regression models (9.3 %, 4 articles) (Table S1). The low utilization rate of regression models may be due to the difficulty in obtaining presence/absence data of target species, which these models require [39,45,46]. Compared to regression models, ENMs only require data on the activity points of target species, which improves operability and reliability. Additionally, ENMs reduce the subjectivity inherent in mechanism models, making them the preferred choice (Table S1). The Maxent model, known for its high reliability [54], is the most widely used for evaluating giant panda habitats, selected in more than half of the studies (58.1 %). However, the niche model represented by the Maxent model has poor interpretability and is not conducive to the protection and planning of giant panda habitats [45].

Traditional mechanism models still accounted for a large proportion (34.9 %, 15 articles) in HSE research. Among these, studies using mechanism models (16.3 %, 7 articles) adopted the AHP method to assign different weights to different indicators, partially overcoming the excessive subjectivity of traditional mechanism models [28,55]. However, some studies (16.3 %, 7 articles) assigned equal weights to each factor and merely superimposed the scores of each factor to obtain a habitat suitability distribution map [37,56]. But significant differences have been observed between the habitat selection of wild giant pandas and the traditionally believed suitable habitat conditions [57]. Moreover, many studies using mechanism models did not verify the effectiveness of models based on the actual distribution points of giant pandas, thus indicating a lack of scientific validation [28,55]. Therefore, the rationality of the method requires further consideration.

A comparison of the above advantages and disadvantages shows that the accuracy of the comprehensive application of the AHP and Maxent models is improved compared to that of a single model [45]. Model accuracy is also influenced by the spatial range and index granularity of the evaluation, for which an optimal interval exists, maximizing the model's evaluation accuracy [58]. Further research should explore the advantages and disadvantages of different models, provide detailed and scientific data analyses, and combine various technologies to obtain more accurate results.

#### 4.2.3 Indicator selection

The indicators selected to evaluate giant panda habitat quality are still relatively narrow, focusing mainly on four categories: topography (95.3 %, 41 articles), vegetation (88.4 %, 38 articles), human interference (76.7 %, 33 articles), and food resources (bamboo) (53.5 %, 23 articles) (Fig. 3). However, factors such as natural interference and climate, which significantly impact on giant panda habitats, were rarely included in the assessment.

For biological factors, many studies only evaluated vegetation and food resource quality in habitats by indicators such as the vegetation cover type (74.4 %, 32 articles) and bamboo species distribution (44.2 %, 19 articles). They merely graded and scored different vegetation cover types (coniferous forest, mixed coniferous and broad-leaved forest, shrubs, and farmland) and bamboo species (based on giant panda preferences) according to human experience. Thus, a more detailed analysis is lacking, and the results may not be satisfactory. Hull et al. (2014) [29] reviewed research on habitat selection for giant pandas and analysed 46 habitat factors. The results revealed significant correlations between microenvironmental factors, such as tree diameter, shrub height, shrub coverage, bamboo coverage, and habitat selection of giant pandas. However, these factors were often ignored in large-scale HSE studies. Kang et al. (2017) [59] compared the habitat characteristics of artificial and natural forests in the Wanglang Nature Reserve, finding that artificial forests had excessively high tree density, lacked bamboo resources, and had significantly lower biodiversity levels in the tree and shrub layers than natural forests. Artificial forests cannot meet the ecological needs of giant pandas. Habitat differences between artificial and natural forests are difficult to detect with current giant panda habitat suitability evaluations. Thus, future studies should incorporate more microenvironmental indicators, such as bamboo density, which play a crucial role in determining core protected areas [60].

For human interference, most studies only quantified this factor based on the distance from residential areas (39.5 %, 17 articles) and roads (72.1 %, 31 articles) (Table S1). The Fourth National Giant Panda Survey recorded 17 types of human activity interference factors, with grazing and transportation roads posing the greatest threat [10]. However, few studies have evaluated how these disturbances affect the quality of giant panda habitats. More in-depth analyses of human interference impacts on wildlife habitats at a detailed scale are necessary [61]. In addition, socioeconomic factors, which directly or indirectly impact wildlife habitat quality, have not been assessed in current HSE studies [62].

The main distribution area of giant pandas is also an earthquake-prone region, where earthquakes and secondary disasters, such as landslides and debris flows, can severely damage forest ecosystems and threaten the survival of giant pandas [31]. According to the Fourth National Giant Panda Survey, the Wenchuan earthquake directly damaged 57,800 hm^2^ of giant panda habitat, accounting for 2.5 % of the total habitat area in China [63]. Therefore, natural interference factors must be considered when evaluating areas with frequent geological disasters. However, few current studies have included natural interference factors, such as earthquakes, in their evaluations (Table S1).

We found that it is worth noting that some studies have evaluated habitat suitability of giant pandas and other species in the same domain (such as takin) using the same indicators to explore the symbiotic relationship between them [24,25,64]. In fact, for evaluating habitats of large terrestrial mammals, similar indicators are often employed. Factors such as terrain and human interference can also be applied to habitat evaluation studies of other large mammals [65]. Due to the highly specialized feeding habits of giant pandas, bamboo quality is often used as an indicator to evaluate their food resource quality when evaluating their habitat quality [28,49]. Similar to giant pandas, red pandas are highly specialized vegetarian carnivores that mainly feed on bamboo too [66]. However, research on habitat evaluation for red pandas is currently limited, and bamboo has not been utilized as an indicator for assessing food resource quality in their habitats [67]. We believe that the methodologies of HSEs developed for giant pandas hold significant reference value for similar studies on other endangered mammals such as red pandas in the same domain.

Overall, the limitations imposed by the narrow indicators used in HSE studies have greatly affected the accuracy of the evaluations. The basic attributes of ecosystems can be divided into three categories: composition, structure and function [68]. Currently, indicators for evaluating giant panda habitats primarily focus on ecosystem structure, with scant consideration given to ecosystem composition and function. Moreover, the current evaluation indicators cannot effectively assess habitat quality at a microenvironmental scale. Numerous studies have confirmed that micro environmental indicators such as the height, density of trees, shrubs, and herbaceous layers can affect the habitat selection of giant pandas, yet these indicators are rarely used in habitat evaluation studies [57,60,69]. While the importance of social and economic factors in wildlife conservation is acknowledged, research on integrating socioeconomic factors into habitat suitability assessments remains scarce [70]. Future research efforts should therefore expand the scope of evaluation indicators and conduct habitat suitability evaluations on a more refined scale. In addition, the complexity of habitat selection for giant pandas has increased because of interactions between different habitat characteristics, which is also applicable to HSE research on giant pandas. Future studies aim to analyze these interaction effects, which are pivotal for understanding habitat dynamics [29].

### 4.3 Implications for giant panda protection

Early studies were constrained by limited indicator selection, data availability, high subjectivity, and focused research areas. Consequently, these studies could only assess habitat suitability in a few mountain ranges or protected areas, failing to comprehensively explore landscape-scale habitat distribution patterns or factors such as climate change affecting giant panda conservation through HSE technology. Therefore, it was also difficult to propose scientific protection suggestions [14,34]. As technology has advanced, HSE studies for giant pandas are evolving towards greater diversity. This evolution enables the determination of suitable habitat distributions for giant pandas and provides critical guidance for their conservation efforts.

Overall, with the continuous protection efforts of the Chinese government, the habitat area and quality of giant pandas are steadily increasing. Numerous habitat suitability evaluations (HSEs) have identified human interference as a primary threat to giant panda habitats [17,28]. If human interference can be effectively controlled, many degraded habitats can naturally recover [38]. Some artificial measures can be taken to restore degraded habitats. We found that the distribution results of habitat quality for the same region did not differ significantly from that in earlier studies [14,18,39,44]. It is interesting to note that some studies suggest that in the context of global warming, the suitable habitat area for giant pandas will increase by around 2050 compared with now and decrease by around 2100. To some extent, global climate change may not have too many negative impacts in the short term [23,71]. However, these studies only evaluated the ecological habits of giant pandas and did not fully consider the habitat distribution of bamboo and other species in the same domain after being affected by climate change. These biological factors will undoubtedly affect the habitat distribution of giant pandas, so relevant researchers should be cautious about this conclusion. In addition, studies have shown that there are significant differences in the evaluation results when factors such as human interference and climate change are selected in the evaluation factors or not, indicating that different evaluation criteria have a significant impact on the evaluation results [38,48].

Therefore, we propose expanding the scope of evaluation indicators in the future research to develop a comprehensive and universally applicable index system for effectively assessing the quality of giant panda habitats. The distribution of giant panda habitats in a large number of regions shows a patchy and fragmented trend, and habitat protection work should focus on the habitat corridor. Studies on giant panda habitat suitability offer theoretical insights into identifying corridor locations, prioritizing areas for habitat restoration, and setting restoration goals [28,39,44,72]. Currently, only Currently, only specific conservation management areas adequately support giant panda survival, with many suitable habitats lying outside these managed zones. Moreover, under the influence of climate change, human interference, and other factors, the current management patterns of protected areas may not be suitable for future protection work [19,71]. Therefore, future studies should broaden the scope of protection management, adjust the internal management level of protected areas, and strengthen patrol monitoring [25,26.51].

## 5 Conclusion

We conducted a systematic review of HSE studies for giant pandas published in Chinese and English journals between 2013 and 2022 and, identifying and analyzing 43 relevant papers. Our analysis focused on research scales, evaluation methods, and research results. Overall, the geographical distribution of giant panda HSE study areas was consistent with the geographical distribution of wild giant pandas. Compared to earlier studies, current HSE-based research on giant pandas has advanced significantly in terms of application breadth and evaluation accuracy and is developing at a large scale and in an interdisciplinary direction. However, shortcomings still remain, including the concentration of studies in specific regions, incomplete evaluation indicators, and reliance on outdated data. A universally effective method for assessing habitat suitability remains elusive. We suggest that future research should broaden the application of HSE technology, particularly in the management and design of the Giant Panda National Park, and pay more attention to areas that either have not been evaluated or have not been thoroughly evaluated. Giant panda HSEs can offer researchers valuable insights for determining suitable habitat distributions and informing strategies for habitat protection, management planning of protected areas, and other conservation initiatives. Future evaluations should carefully weigh the strengths and limitations of various modeling approaches, enhance model accuracy, expand evaluation criteria, incorporate finer-scale analysis of microbiological indicators, and ensure timely updates of databases.

Future conservation efforts should effectively control human interference in giant panda habitats and artificially restore degraded habitats. Habitat protection should focus on habitat corridors, the scope of protection management should be expanded, the internal management level of the protection zone should be adjusted promptly, and patrol monitoring should be strengthened. Current evaluations of the suitability of the giant panda habitat can inform large-scale habitat restoration efforts, contribute to the development of the Giant Panda National Park, and protection of same-domain species in the future. Recent studies have demonstrated the effectiveness of giant panda conservation in China. Our review summarises the latest progress in HSE-based research on giant pandas and provides detailed statistics on the research scale, evaluation methods, and results of HSEs. This review addresses the existing gap in systematic reviews of HSE-based research on giant pandas. Moreover, this work provides suggestions for future HSE-based research on giant pandas and the protection and management of giant panda habitats.

## Funding

This study received financial support from the 10.13039/501100001809National Natural Science Foundation of China grant 32470535 (to B.Y.) and the Major Science and Technology Projects of the 10.13039/501100004829Science and Technology Department of Sichuan Province grant 22ZDYF2289 (to B.Y.)

## Ethics approval

This review article does not involve any human participants or animal experiments, and therefore does not require approval from an ethics committee.

## Data availability statement

Data associated with our study has not been deposited into a publicly available repository. All data needed to generate the results are provided in the Supporting Information files.

## CRediT authorship contribution statement

**Guanyu Mu:** Writing – review & editing, Writing – original draft, Visualization, Validation, Supervision, Methodology, Formal analysis, Data curation, Conceptualization. **Xiaotong Shang:** Writing – review & editing, Methodology. **Han Pan:** Writing – review & editing, Methodology. **Tao Ruan:** Writing – review & editing, Visualization. **Biao Yang:** Writing – review & editing, Writing – original draft, Supervision, Methodology, Formal analysis, Conceptualization. **Li Zhang:** Writing – review & editing, Writing – original draft, Methodology, Conceptualization.

## Declaration of competing interest

The authors declare that they have no known competing financial interests or personal relationships that could have appeared to influence the work reported in this paper.
